# Nest success of ground‐nesting ducks in the wetlands of Great Salt Lake, Utah

**DOI:** 10.1002/ece3.10384

**Published:** 2023-07-28

**Authors:** Mark E. Bell, Michael R. Conover

**Affiliations:** ^1^ Department of Fisheries and Wildlife Michigan State University East Lansing Michigan USA; ^2^ Department of Wildland Resources, Ecology Center Utah State University Logan Utah USA

**Keywords:** cinnamon teal, gadwall, Great Salt Lake, mallard, nearest‐neighbor distances, nest concealment, nest predators, nest success, raccoons, skunks

## Abstract

The number of ground‐nesting ducks in the wetlands of Great Salt Lake, Utah has drastically decreased in the past few decades. A potential cause for this decline is the increase of predator species and their abundances, which has caused most nests to fail from depredation. Ground‐nesting ducks may be able to reduce the risk of nest depredation by selecting nest sites where local physical structures or vegetation provides olfactory or visual concealment. To test this, we used logistic exposure models to look at the effect of nest‐site characteristics on daily survival rates (DSRs) of nests during 2019, 2020, and 2021 in the wetlands of Great Salt Lake, Utah. We found 825 duck nests including 458 cinnamon teal (*Spatula cyanoptera*), 166 mallards (*Anas platyrhynchos*), and 201 gadwalls (*Mareca strepera*). DSRs were 0.9714 ± 0.0019 in 2019, 0.9282 ± 0.0049 in 2020, and 0.8274 ± 0.0185 in 2021. Survival rates varied among years but not among duck species. Striped skunks (*Mephitis mephitis*) and raccoons (*Procyon lotor*) were responsible for 85% of depredated nests. Nests located near other duck nests had higher DSRs than more dispersed nests. Neither visual nor olfactory characteristics correlated with increased DSRs based on AIC_*c*_ analysis. Nests located inside a mixed nesting colony of American avocets (*Recurvirostra americana*), black‐necked stilts (*Himantopus mexicanus*), and common terns (*Sterna hirundo*) had higher DSRs than duck nests outside the colony. Increased nesting densities of ducks and other colonial waterbirds had the greatest impact on nesting success. Increased nest density may be encouraged through early spring green‐up.

## Graphical abstract

Nesting success of ducks in the wetlands of Great Salt Lake, Utah has drastically reduced in the last few decades. Increased species richness and abundance of predators overwhelms nesting ducks, and most nests fail.

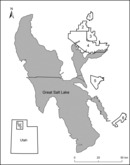

## 1 INTRODUCTION

The greatest risk posed to the survival of duck nests during incubation is nest depredation (Sargeant & Raveling, 1992; Walker et al., 2013) from both avian predators (Bruggink et al., 1994) and mammalian predators (Klett & Johnson, 1982; Walker et al., 2005). Ground‐nesting ducks attempt to hide their nests from visual detection as a means of lessening depredation risk. For birds using this strategy, the selection of a nest site is one of the most important choices a bird makes in protecting its nest from predators (Borgo & Conover, 2016b; Dyson et al., 2019; Klett et al., 1988; Walker et al., 2005). Nest sites with high levels of lateral concealment (obstruction from side views at ground level) have increased protection from detection by terrestrial predators (Borgo & Conover, 2016b; Setash et al., 2020). Tall vegetation surrounding nest sites provides overhead concealment and correlates with lower levels of nest depredation (Albrecht & Klvaňa, 2004). More nests are successful in hatching eggs when they are located in large patches of tall vegetation, have tall neighboring shrubs bordering the nest, and are surrounded by vegetation with high levels of species richness (Cowardin et al., 1985; Crabtree et al., 1989).

Many mammalian predators hunt at night and use olfaction more than vision to locate nests. Surface features, such as a boulder or tall clump of vegetation, that protrude into the air or a rough vegetation canopy increase atmospheric turbulence close to the ground (Borgo & Conover, 2016b; Conover, 2007), and greater turbulence intensity makes it more difficult for olfactory predators to find nests. In the Prairie Pothole Region, duck nests with more atmospheric turbulence (more change in wind direction and velocity) had higher daily survival rates (DSRs) than other nests (Borgo & Conover, 2016b), but ducks did not select for this trait when searching for a nest. Additionally, weather variables that influence olfactory detection, specifically precipitation and high humidity, correlate positively with daily nest survival (Fogarty et al., 2017, 2018).

Non‐vegetative factors, such as high nest density, can also improve nest survival. Mallard (*Anas platyrhynchos*) and gadwall (*Mareca strepera*) nests at Suisun Marsh, California were more successful when located near neighboring duck nests (Ringelman et al., 2014). This would especially be true when duck nests are so plentiful that the local predators cannot eat all the eggs, a phenomenon called predator swamping (Conover et al., 2005). Nesting by others may also be beneficial by diluting the risk of any individual nest being depredated. Other studies, however, reported that nest density did not affect nesting success (Ackerman, 2002; Padyšáková et al., 2011).

Freshwater wetlands created by impounding rivers flowing into Great Salt Lake (GSL), UT support many waterfowl species throughout the year. Thousands of ducks arrive in spring to court and nest, remaining until fall when they migrate to winter ranges. In North America, most ducks nest farther north in the Prairie Pothole Region of Canada and northern United States (Greenwood et al., 1995). However, the wetlands of GSL are significant for the world's population of cinnamon teal (*Spatula cyanoptera*), with GSL wetlands being the heart of their breeding range (Baldassarre, 2014; Conover & Bell, 2020). Historically, GSL wetlands produced hundreds of thousands of ducks annually. Bellrose (1980) reported that GSL marshes were used for nesting by tens of thousands of ducks from nearly a dozen species, and that cinnamon teal were the most abundant species with 150,000 ducks present in the GSL area. Bellrose also reported 65,000 gadwalls and 55,000 mallards in the GSL area. Only 34 years later, Baldassarre (2014) reported a drastically lower number of ducks nesting in Utah including 22,000 cinnamon teal, 11,300 mallards, and 8600 gadwalls.

One reason for the decline in waterfowl production on GSL marshes is that GSL flooded during the 1980s, reaching a historic high of 1284 m above sea level during 1987, which was a gain of 4 m above normal levels (Aldrich & Paul, 2002). These floods pushed GSL's saline water into the freshwater and brackish marshes along the edges of the GSL, destroying dams and impoundments and killing nesting vegetation. During these years of floods, ducks that formerly nested on GSL wetlands moved farther north to nest (Foote, 1989). After the GSL shore receded, the dams and impoundments were restored, but it took years before the marsh vegetation returned to its former condition (Aldrich & Paul, 2002). Small number of ducks began to nest in the restored areas, but raccoons (*Procyon lotor*) and red foxes (*Vulpes vulpes*), which were previously absent, had moved into the wetlands (Frey, 2005; West, 2002). Prior to the flood, the striped skunk (*Mephitis mephitis*) was the only major predator of duck nests (Crabtree et al., 1989). So many waterfowl nested in the GSL wetlands prior to the flood that the local skunk population could only eat a small fraction of the eggs. This created a predator swamping effect, allowing the remainder to hatch. After the flood, the population of skunks, raccoons, and foxes were so great that the few duck nests were overwhelmed with predators, and few survived (Frey, 2005; West, 2002). This discouraged ducks from returning to nest in GSL marshes, and duck nest densities never reached the level required to satiate the predators' appetite for duck eggs. GSL wetlands no longer fledge the large number of ducks that they had been prior to the flood (Bell, 2022).

Another possible cause for the decline of duck numbers in the GSL wetlands is the change in vegetation and habitat availability. After the flood of the 1980s ended and the saline waters receded from the wetlands, bare ground was left exposed. Non‐native species colonized these open areas and outcompeted native vegetation (Kettenring et al., 2020). The wetlands today are dominated by non‐native phragmites (*Phragmites australis*). Phragmites stands are so dense that they provide little benefit to waterfowl and are generally unused for foraging or nesting by waterfowl species (Pendelton et al., 2020).

In our study, we examine current nesting success of duck nests in GSL wetlands compared to current vegetation and predator compositions. We hypothesized that ducks would have higher nest success in areas that provide high levels of visual concealment and olfactory concealment. We also hypothesized that nests near other duck nests would have higher nesting success (Ringelman et al., 2014). We also lack information about which predators depredate nests in GSL marshes and their relative abundance in GSL marshes. Hence, we used nest cameras to determine which predators depredated nests. We assessed relative predator abundance using nest cameras set at bridges, on the dams at bait stations, and spotlight surveys at night to determine (1) if ducks avoid nesting in areas where predators are abundant and (2) if ducks that do nest in these areas are less successful than ducks that nest were predators are less abundant.

## 2 STUDY AREA

**FIGURE 1:**
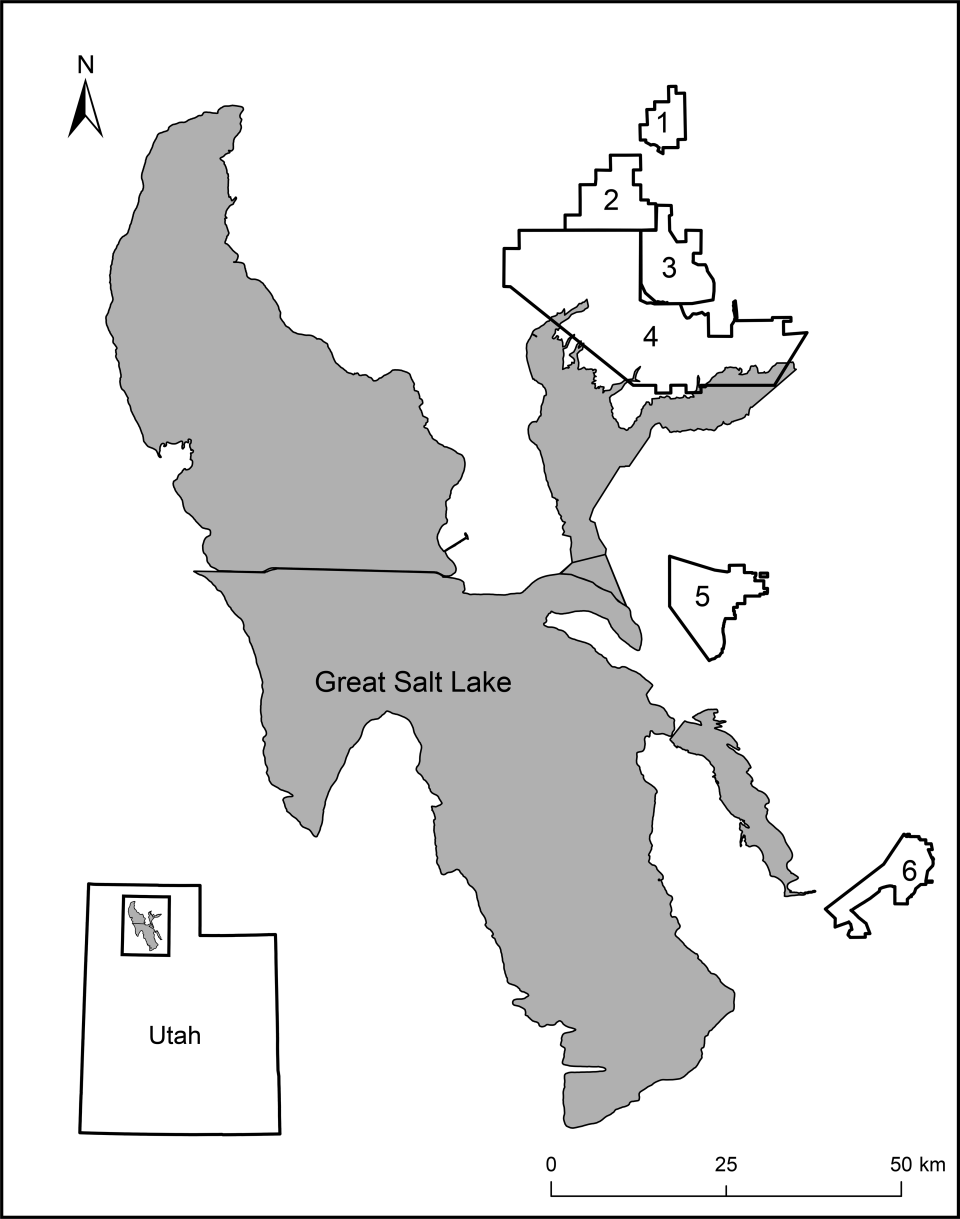
Study sites were located along the eastern shore of Great Salt Lake in northern Utah in Waterfowl Management Areas (WMA). Shown on the map are (1) Salt Creek WMA, (2) Public Shooting Grounds WMA, (3) Bear River Duck Club, (4) U.S. Bear River Migratory Bird Refuge, (5) Ogden Bay WMA, (6) Farmington Bay WMA.

Our study occurred during 2019, 2020, and 2021 in the wetlands bordering GSL, Utah (Figure 1). Utah Division of Wildlife Resources managed several waterfowl management areas (WMAs), which provided habitat for thousands of ducks during the spring, summer, and fall. We located and monitored nests at four WMAs: Salt Creek, Public Shooting Grounds, Ogden Bay, and Farmington Bay. Nests were also located and monitored at U.S. Bear River Migratory Bird Refuge managed by the U.S. Fish and Wildlife Service (hereafter, BRMBR) and the Bear River Duck Club. We refer to all these areas collectively as management areas. They all dammed freshwater flowing towards GSL to make shallow impoundments for waterfowl and waterbird use. The dams that created these impoundments averaged a height of 1 m above the water and were 15–20 m wide with a dirt road running along the center. The total length of dams present at BRMBR was 321, 88 km at Ogden Bay WMA, 66 km at Farmington Bay WMA, 29 km at Public Shooting Grounds WMA, and 20 km at Salt Creek WMA. Impoundments were adjacent to each other, separated only by narrow dams. This was intended to create a maximum amount of flooded area within the management areas. Because of this, dams were the only land that was dry during years with high levels of snowfall. Dams were constructed so that both sides of the dams had a gentle slope from the water's edge up to the road. These sloping sides were covered by grass and forbs and provided nesting habitat for ducks, other waterfowl, and waterbirds; trees did not occur on the dams. Ducks used to nest within the shallower parts of impoundments (Williams & Marshall, 1938), but few ducks nested there during recent years because these areas are now overgrown with phragmites (M. Conover, unpublished study). Each management area conducted predator control from March through May in the form of trapping and lethal removal.

## 3 METHODS

We searched the dams of these management areas for nests every 14 days from May until August. As recommended by Gloutney et al. (1993), searches were conducted between 09:00–15:00 h to check status of old nests and locate new nests. In 2019, we searched a total of 55 km of dam, including 26 km at BRMBR, 14 km at Farmington Bay WMA, 6 km at Public Shooting Grounds WMA, and 9 km at Salt Creek WMA. In 2020, we searched a total of 77 km of dam, including 26 km at BRMBR, 20 km at Farmington Bay WMA, 21 km at Ogden Bay WMA, and 10 km at Public Shooting Grounds WMA. In 2021, we searched a total of 103 km, including 40 km at BRMBR, 20 km at Farmington Bay WMA, 14 km at Ogden Bay WMA, 10 km at Public Shooting Grounds WMA, 9 km at Salt Creek WMA, and 10 km at the Bear River Duck Club. We increased dams searched during 2021 to locate more nests as few ducks nested in the wetlands that year.

Nests were located using a modified chain‐dragging method (Klett et al., 1986). A boom was constructed out of lumber (commonly called 2 × 4 boards), which extended out one side of a pickup bed for 4–6 m. We searched one side of the dam as we drove along the transect, and searched the opposite side as we came back up the starting point. The length of boom was adjusted to accommodate the width of dams being searched. Chains were attached to the boom spaced approximately 25 cm apart. These chains were 8–12 m in length and dragged straight behind the boom as the truck drove along the dam at 10–15 km/h. The chains flowed over and through the vegetation without creating a noticeable disturbance to the cover or nests. As the chains passed over or close to the nests, the incubating hen would flush, allowing an observer in the back of the pickup to locate the nest and identify the species of duck. The eggs were lower than the top of the nest bowl so that the chain did not touch them; only one egg was crushed by the chain during this study. We selected this sampling method because it had been previously employed in these same impounded wetlands (Crabtree et al., 1989; Crabtree & Wolfe, 1988), but the drawback of this sampling method is that it prevented us from sampling any duck nests that were out in the impoundments. However, our searches indicated that few ducks nested there due to phragmites and high water levels in 2019.

Once located, we recorded the GPS location, and each nest was given a unique number and marked by placing two survey flags on the opposite side of the road to aid in relocating the nest for monitoring purposes. An inconspicuous marker was placed on the ground and under the vegetation next to the nest, which assisted in relocating the nest after nesting was completed. We kept our time at nests brief to allow the hen to return quickly. The species of nest was determined by identifying the duck species that flushed from the nest. We recorded the clutch size, and an egg was randomly selected from the nest to determine its age using the floatation method (Brua & Machin, 2000).

Each nest was revisited every 2 weeks to measure clutch size and determine its fate; nests were checked at the same time the dam was searched for new nests. Fate was recorded as successful, depredated, or abandoned by observing egg remains (Klett et al., 1986). Missing, scattered, or broken shells with membranes attached to the shell indicated a depredated nest. Eggs with membranes separated from the shell characterized successful nests. A nest was considered successful if ≥1 egg hatched. A nest was considered abandoned if the eggs appeared undisturbed by a predator and were both cold and unattended by a hen for >1 consecutive visit to the nest.

Game cameras (Cuddeback 20 Megapixal IR, Cuddeback, De Pere, Wisconsin) were installed at approximately 30 nests annually to observe the nest following the methods of Croston et al. (2018), Kruger et al. (2018), and Blythe and Boyce (2020). Cameras were placed approximately 0.5 m away from the nest. Height of the cameras was adjusted to place the top of the camera below the surrounding vegetation to decrease visual clues to predators of nest locations. Camera images were used to confirm if a nest was successful or depredated, and used to identify the species of predator that depredated the nest.

Vegetation surveys began on July 16, 2019, July 20, 2020, and July 19 in 2021. We waited to conduct vegetation surveys following the completion of all nests to reduce disturbing incubating hens and potentially influencing nesting outcomes. The delay may adversely impact results as vegetation changes over time (McConnell et al., 2017; Ringelman & Skaggs, 2019), but we felt this risk was less serious than the risk that a disturbance during incubation would have on reducing nesting success. The predominant plant species in the vegetation patch surrounding each nest was determined by tallying the species found at four points 1‐m from the nest in each of the cardinal directions. If a species was found at ≥2 of the four points surrounding the nest, it was considered the predominant species. If there were two species found in equal amounts then the surrounding vegetation was considered a mixed stand of the two species.

At each nest, we recorded eight variables: mean height and standard deviation of vegetation in the patch surrounding the nest, overhead concealment and lateral concealment at the nest site, dam width, tallest plant adjacent to the nest, width of the dam, distance from the nest to the edge of the road, and distance to the nearest duck nest. We defined the nesting patch as the circular area within 10 m of the nest. To determine the mean height and roughness of the vegetation in the nesting patch, we ran a transect perpendicular to the dam starting at the edge of the road and running to the water's edge and a second 20‐m transect parallel to the dam. Both transects ran through the nest, and the parallel transects were located so that their midpoint was at the nest site. At 1‐m intervals along the transects, we measured the height and species of the vegetation (Fogarty et al., 2017). Vegetation heights along the two transects were used to determine the height mean and SD of vegetation in the nesting patch; the latter was used as a metric of surface roughness (Conover, 2007). The surface roughness of vegetation within the nesting patch was used as an index of olfactory concealment because a rough upper surface causes atmospheric turbulence. The tallest plant adjacent to the nest was measured using a meter stick because a tall plant that extends above the canopy will also cause turbulence (Conover, 2007).

Overhead concealment was measured by placing a 20 × 20 cm square with a checkerboard pattern made up of 4 × 4 cm squares in the nest bowl. Looking down from a height of 1.5 m, an observer counted how many of the squares' corners were obstructed from view by overhead vegetation; the proportion hidden from view was used as a measure of concealment (Borgo & Conover, 2016b). We assessed lateral concealment by placing a 2‐m tall pole marked in dm in the center of the nest. An observer recorded the minimum height at which the pole was visible to them from a distance of 4 m away from the nest and with their eyes at a height of 1 m in each of the 4 cardinal directions (Bruggink et al., 1994; Fogarty et al., 2017; Ringelman & Skaggs, 2019). We measured the dam width from the center of the road along the top of the dam to the water's edge on the side of the dam where the nest was located. Distance from road was how far the nest was from the edge of the road. We obtained monthly precipitation measurement for March, April, and May of each year from the Brigham City weather station, which was the closest one to the management areas (National Oceanic and Atmospheric Administration, 2021).

We surveyed predators using spotlights at BRMBR during the night once a week for the 3 weeks prior to our nest searches in 2020 and 2021, resulting in three predator counts for each year. We conducted the spotlight survey along the same 26‐km transect of where we would later conduct nest searches. Surveys began 30 to 60 min after sunset and continued until completion, which was usually around midnight. We drove along the transect at 15 km/h with two technicians in the bed of the pickup. Each technician searched one side of the dam and was equipped with a spotlight (Energizer) that allowed us to identify predators up to 50 m away. A predator was recorded only when we drove past it to avoid double counting of the same predator. Each time a predator was found, we recorded the GPS location, species, and number of predators.

As a second method to assess relative predator abundance, we installed the same game cameras used for nest monitoring at each of the 15 bridges along the dams where we conducted our spotlight surveys. We monitored predators at bridges during the same 3 weeks in which we conducted spotlight surveys. Each bridge was an average of 1.5 km apart from the nearest bridge (ranging 0.1 to 3.4 km). Bridge cameras were used to record the total number of predators observed nightly crossing any bridge and averaged over a 1‐week period. Sightings of the same predator species at the same bridge that occurred within 2 min of each other were considered the same individual and were only counted once. This resulted in three data points each year given the 3 weeks before the nesting season. Bridge camera activity was an index of relative abundance of predators at bridges along the dams.

Additionally, during the 3 weeks prior to nest searches, we set up 10 bait stations at mid‐points between bridges along the main dams. Each bait station was an average of 2.8 km from the nearest bait station (range 1.6 to 5.7 km). At each bait station, we poked holes in the top of a can of tuna fish, staked it to the ground, and installed a game camera to observe the site. Sightings at a bait station of the same predator species that occurred within 2 min of each other were considered the same individual and were only counted once. We placed fresh cans of tuna each week to keep the scent present at each of the bait stations. Cameras were inspected at the bridges and bait stations once a week for function and proper battery levels. The memory cards were switched out weekly to avoid reaching capacity. Bait station data were recorded as the number of predator visits per bait station per night and averaged over a 1‐week period, yielding three weekly data points each year. Activity at bait stations reflected the relative abundance of predators present along the main dams. We created an index of relative predator abundance for each impoundment by adding the predator averages for each impoundment from the bridge, bait stations, and spotlight counts.

After all nests were located, we calculated the distance from each nest to the nearest neighboring duck nest. Successful nests may have shorter nearest‐neighbor distances than unsuccessful nests either because nests that are close together may be less likely to be depredated due to (1) a dilution effect and predator swamping or (2) some feature in the environment that affords duck nests some protection from predators and attracts nesting ducks. Such a feature may be a large colony of American avocets (*Recurvirostra americana*), black‐necked stilts (*Himantopus mexicanus*), and common terns (*Sterna hirundo*) that occurred along a 6.4‐km stretch of dam at BRMBR (hereafter called the colony). These birds nested on a series of small (<50 m^2^) low‐lying islands within a few meters of the dam and on the dam adjacent to these islands. Ideally, we would have accounted for this by calculating the distance of the nearest neighboring nest of any species, but our search method was not effective at locating the nests of these colonial birds as they leave their nests prior to the chains passing over them. Rather, we recorded whether a duck nest was located inside or outside of the colony to test for effects on nest survival.

We calculated the density of duck nests for each of the nine impoundments in BRMBR during 2020 and 2021, giving us a sample size of 18 impoundment‐years (2 years × 9 impoundments). We determined nest density after correcting for nests that were depredated before we could find them using the methods of Arnold et al. (2007). Area was determined by multiplying the length of the dam that we search along the impoundment by the mean width of the dam along the same impoundment. We assigned each nest the value of nest density and relative predator abundance from the impoundment on which it was located. We determined whether each nest was located within the colony or outside of the colony and grouped them accordingly. We assigned the incubation day and vegetation type surrounding the nest to each nest.

### 3.1 Statistical analysis

We used a Pearson correlation test in Program R (version 4.2.1; R Core Team, 2022) to evaluate for collinearity among variables (those with *R* > 0.20). We fit an initial logistic exposure model using Program MARK (White, 2001) to analyze DSRs and then ran an additional models testing the combined effect of every combination of variables that were not collinear. We assessed model parsimony using the second‐order Akaike's Information Criterion (AIC_c_). Models were considered competitive if the ΔAIC_c_ was <2 from the top model (Arnold, 2010). We subset the nests to include those only found in BRMBR and ran a model testing the effect of being located inside the colony of avocets, stilts, and terns had on DSRs. We then further subset the nests to include those found in 2020 and 2021 at BRMBR and ran an additional model testing the effect of predator abundance and nest density on DSRs.

## 4 RESULTS

We found a total of 825 duck nests, including 458 cinnamon teal, 166 mallards, and 201 gadwalls. We located 458 nests in 2019, 240 in 2020, and 127 in 2021. From our initial analysis, we found the DSRs to be 0.9714 ± 0.0019 (DSR ± SE) for 2019, 0.9282 ± 0.0049 for 2020 and 0.8274 ± 0.0185 for 2021. We found a DSR of 0.9614 ± 0.0024 for cinnamon teal, 0.9601 ± 0.0037 for mallard, and 0.9525 ± 0.0044 for gadwall from all years combined. Spring precipitation receive from March through May varied across the years with 24 cm of precipitation received in 2019 compared to 4 cm of precipitation during the same time period in 2020 and 2021.

Across all 3 years, 71 predators were recorded on camera while depredating nests. We found 30 striped skunks, 31 raccoons, three long‐tailed weasels (*Mustelidae frenata*), two Sandhill cranes (*Antigone canadensis*), two northern harriers (*Circus hudsonius*), one red fox, one coyote (*Canis latrans*), and one California gull (*Larus californicus*). Skunks were responsible for 46% of all depredated nests and raccoons for 39% of nests.

Our top DSR model for nest‐site characteristics included width of dam and distance to nearest neighbor. Nests located along narrower dams ($\hat{\beta }$ = −0.07 + 0.2, $\hat{\beta }$ + SE) and closer to neighboring nests ($\hat{\beta }$ = −0.001 + 0.0002) had higher DSRs. There were three other models that were within two AIC_c_s from the top model. The first competitive model included width of dam, vertical concealment, and distance to the nearest neighbor. Nests on narrower dams ($\hat{\beta }$ = −0.07 + 0.02) and near neighboring nests ($\hat{\beta }$ = −0.001 + 0.0002) had higher DSRs, but the confidence interval of vertical concealment ($\hat{\beta }$ = −0.2 + 0.2) overlaps 0, so it is likely an uninformative variable (Arnold, 2010). The second competitive model included width of dam, distance from road, and distance to nearest neighbor. Nests on narrower dams ($\hat{\beta }$ = −0.07 + 0.02) and near neighbors ($\hat{\beta }$ = −0.001 + 0.0002) had higher DSRs but the confidence interval of distance to road ($\hat{\beta }$ = 0.04 + 0.04) bounded 0, showing that this parameter was uninformative. The third competitive model included width of dam, vertical concealment, the standard deviation of vegetation height, and the distance to nearest neighbor. Nests on narrower dams ($\hat{\beta }$ = −0.07 ± 0.02) and close near neighbors ($\hat{\beta }$ = −0.001 + 0.0002) had higher DSRs but vertical concealment ($\hat{\beta }$ = −0.2 + 0.2) and the standard deviation of vegetation height ($\hat{\beta }$ = −0.2 + 0.2) bounded 0 and were considered uninformative parameters. No other models were competitive in this AIC_c_ analysis (Table 1).

**TABLE 1:** Model selection for impact of nest‐site characteristics on DSRs for all duck species found during 2019, 2020, and 2021 in Great Salt Lake wetlands using AIC_c_ weights (*w*_*i*_) to determine which variable(s) were most strongly associated with the DSRs of nests.

Model	*K*	AIC_c_	ΔAIC_c_	*w*_*i*_	Loglik
Nearest neighbor + dam width	3	1202.8	0.0	0.33	−598.4
Nearest neighbor + dam width + vertical concealment	4	1203.5	0.7	0.23	−597.8
Nearest neighbor + dam width + distance from road	4	1204.0	1.2	0.18	−598.0
Nearest neighbor + dam width + vertical concealment + height SD	5	1204.6	1.8	0.13	−597.3
Nearest neighbor + dam width + distance from road + vertical concealment + height x¯	6	1205.8	3.0	0.072	−596.9
Nearest neighbor + dam width + distance from road + vertical concealment + height SD	6	1206.6	3.8	0.048	−597.3

*Note*: Variables included overhead concealment, lateral concealment, average height of vegetation in the nest patch (height x¯), standard deviation of the height of vegetation in the nest patch (height SD), dam width, tallest plant adjacent to the nest, distance of nest from road, and distance to nearest neighbor. *K* is the number of parameters included in the model. Loglik is the log likelihood of the model. AIC_c_ is the second‐order Akaike's Information Criterion.

Nest densities were 7.5/ha in 2019, 2.9/ha in 2020, and 3.3/ha in 2021, and nest density had a positive effect on DSRs ($\hat{\beta }$ = 0.91 ± 0.07). During 2020 and 2021, we observed 776 predators, including 68 predators (48 skunks and 19 raccoons) using spotlights, 530 predators (151 skunks and 374 raccoons) using bridge cameras, and 178 predators (104 skunks and 72 raccoons) using bait stations. Predator abundance ($\hat{\beta }$ = 0.82 ± 0.05) had a positive association with DSRs.

During 2019, 136 duck nests were located inside the colony and 230 outside it. Nests located inside the colony had higher DSRs ($\hat{\beta }$ = 1.32 + 0.02) than nests located outside the colony. Avocets, stilts, and terns did not nest during 2020 and 2021. With no colony present during those years, we were able to determine whether it was, in fact, the colony that influenced results and not some other feature in the area where the colony was located. During 2020, 12 duck nests were in the area where the colony had been and 116 outside it. The effect of being located within the geographic area of where the colony was the year prior was less than the effect of being in that location when the colony was present ($\hat{\beta }$ = 0.99 + 0.09) but was still positively associated with DSRs. In 2021, the effect of being in that location had a negative effect on the DSRs of duck nests ($\hat{\beta }$ = −0.72 + 0.001).

## 5 DISCUSSION

We found nesting success in the GSL wetlands (29%) was markedly lower than that found by Crabtree et al. (1989, 48%) in these same wetlands, and the number of nests in these areas was far lower during our study than during Crabtree's. While Crabtree et al. (1989) reported depredations by skunks, they made no mention of raccoons. We found that raccoons are now a major predator present in the wetlands.

Nest success varied among years in our study. More nests were successful in 2019 than in 2020 or 2021. We hypothesize that this is due to variation in spring precipitation. In 2019, 24 cm of precipitation were received between March and May. Only 4 cm of precipitation were received during the same time period in both 2020 and 2021. In 2019, there was an early green‐up of vegetation that provided both taller and more dense nesting vegetation for ducks during the nesting season. The early green‐up likely attracted more ducks to remain and nest in GSL wetlands, as well as attracting avocets, stilts, and terns, which increased the nest density and improved nesting success rates. Klett and Johnson (1982) reported a variation of 26% in nesting success for mallards and 21% for blue‐winged teal (*Anas discors*) in the Prairie Pothole Region between wet and dry years, indicating that spring precipitation has been an important factor in nesting success in other areas.

We found that nest success rates were similar among duck species. We were not surprised by this because their nests were interspersed on the dams, had similar characteristics, and were depredated by the same predators. In contrast, other studies reported differences in nesting success rates among duck species interspersed in the same study areas (Klett & Johnson, 1982; Ringelman et al., 2014). We hypothesized that nests would be more successful when located in areas with visual concealment (Albrecht & Klvaňa, 2004; Crabtree et al., 1989; Hines & Mitchell, 1983) and olfactory concealment (Borgo & Conover, 2016a, 2016b; Conover, 2007; Fogarty et al., 2017, 2018). However, we did not find evidence that factors of visual or olfactory concealment improved DSRs.

We found short nearest‐neighbor distances and narrower dams had the greatest impact on DSRs. Ringelman et al. (2014) also found that mallard and gadwall nests at Suisun Marsh, California were more successful when located near neighboring nests. However, other studies found nest density (which is correlated to nearest‐neighbor values) did not affect nesting success, perhaps because nest density did not reach levels high enough to satiate local predators (Ackerman, 2002; Ackerman et al., 2004; Padyšáková et al., 2011).

A second reason why duck nests may be clumped together is that there is some feature that attracts a high density of nesting ducks and also protects them from predators. This feature may be the presence of the avocet, stilt, or tern colony present at BRMBR. These birds emit alarm calls when predators approach their colonies and terns swoop at predators when they come close to their nests. These behaviors may afford some protection to nearby nesting ducks. Furthermore, predators may prefer to prey upon the abundant eggs in these colonies rather than having to search for duck nests. Dwernychuk and Boag (1972), Liordos and Lauder (2015), and Väänänen et al. (2016) also found that nesting success of ducks was higher when nesting near colonial birds, especially small gulls. In 2019, we found that nests inside the colony had higher DSRs than those outside it. During 2020, there was no colony, and we found that DSRs for nests in the area where the colony was located previously were still higher than other locations in 2020, although there was less of a positive effect on DSRs than the previous year. In 2021, nests located in this same area had lower DSRs than other areas in BRMBR. The presence of the colony did improve DSRs of nests in 2019.

Our results reflect a considerable change from earlier studies at BRMBR. Williams and Marshall (1938) checked 1560 nests and reported that 70% of all eggs hatched, 4% were depredated, and 4% abandoned. The remaining 22% of eggs were lost to flooding. The major predator responsible for the few nest depredations was the black‐billed magpie (*Pica hudsonia*), and no mention of fox, raccoon, or coyotes was made. Crabtree et al. (1989) examined gadwall nests at BRMBR during 1983 and reported 48% of nests were successful. Again, no mention of raccoons, but Crabtree et al. identified skunks as the main depredator of nests. During our study, 29% of duck nests were successful, and raccoons and skunks posed a similar risk of depredating duck nests. Raccoons invaded GSL marshes in recent decades (West, 2002, BRMBR unpublished records). Our cameras also photographed weasels, cranes, harriers, and gulls depredating nests on occasion, but not enough for these species to be considered major nest predators. We were surprised to observe cranes and harriers depredating nests. However, harriers were not depredating eggs, rather some of the ducklings had hatched and the harriers were consuming them.

Given the threat that skunks and raccoons pose to duck nests, we hypothesized nests would have higher DSRs in areas of lower predator abundance. However, our data showed that the DSRs were higher in areas with higher relative abundances of predators. Given that mammalian predators can modify their distribution based on prey availability (Ackerman, 2002; Eichholz et al., 2012; Frey & Conover, 2006; Holt, 1977), it is likely, skunks and raccoons in our study area shifted their distribution to take advantage of where ducks were located. Either these areas provided safer nesting areas, or the density of nests in these areas were enough nests to swamp the predators. Our results indicated that nest density is the greatest safety for duck nests in GSL wetlands.

With the steep decline in the duck nesting population of GSL wetlands, focus should be placed on developing methods to help increase nest success by increasing the number of nesting ducks. Bolstering nest success in these wetlands will be the result of increased numbers of ducks nesting here and increased nesting density. Further work should be done to investigate what factors attract ducks to remain and nest in GSL wetlands. Our study suggests that spring growth of vegetation may attract ducks, as well as other colonial waterbirds, and that increased nesting density provides for the highest nesting success rate.

## AUTHOR CONTRIBUTIONS

**Mark E. Bell:** Conceptualization (equal); formal analysis (lead); methodology (lead); writing – original draft (lead); writing – review and editing (equal). **Michael R. Conover:** Conceptualization (equal); funding acquisition (lead); writing – review and editing (equal).

## FUNDING INFORMATION

Great Salt Lake Ecosystem Program; Utah Agriculture Experiment Station; Utah State University Ecology Center.

## CONFLICT OF INTEREST STATEMENT

Neither of the authors have any conflicts of interest in this study. Permission to reproduce materials from other sources (mention “None” if not applicable for your article): None.

## Data Availability

The Data that support the findings of this study are openly available in Dryad at: https://doi.org/10.5061/dryad.9w0vt4bkh
